# CLE-CLAVATA1 Signaling Pathway Modulates Lateral Root Development under Sulfur Deficiency

**DOI:** 10.3390/plants8040103

**Published:** 2019-04-18

**Authors:** Wei Dong, Yinghua Wang, Hideki Takahashi

**Affiliations:** Department of Biochemistry and Molecular Biology, Michigan State University, East Lansing, MI 48824, USA; wdong@msu.edu (W.D.); wangyi64@msu.edu (Y.W.)

**Keywords:** *Arabidopsis thaliana*, CLE peptide, CLAVATA1, root system architecture, small signaling peptide, sulfate, sulfur

## Abstract

Plant root system architecture changes drastically in response to availability of macronutrients in the soil environment. Despite the importance of root sulfur (S) uptake in plant growth and reproduction, molecular mechanisms underlying root development in response to S availability have not been fully characterized. We report here on the signaling module composed of the CLAVATA3 (CLV3)/EMBRYO SURROUNDING REGION (CLE) peptide and CLAVATA1 (CLV1) leucine-rich repeat receptor kinase, which regulate lateral root (LR) development in *Arabidopsis thaliana* upon changes in S availability. The wild-type seedlings exposed to prolonged S deficiency showed a phenotype with low LR density, which was restored upon sulfate supply. In contrast, the *clv1* mutant showed a higher daily increase rate of LR density relative to the wild-type under prolonged S deficiency, which was diminished to the wild-type level upon sulfate supply, suggesting that CLV1 directs a signal to inhibit LR development under S-deficient conditions. *CLE2* and *CLE3* transcript levels decreased under S deficiency and through CLV1-mediated feedback regulations, suggesting the levels of CLE peptide signals are adjusted during the course of LR development. This study demonstrates a fine-tuned mechanism for LR development coordinately regulated by CLE-CLV1 signaling and in response to changes in S availability.

## 1. Introduction

Plant roots optimize nutrient uptake capacity by altering the root system architecture (RSA) in the soil environment [1]. Changes in nutrient availabilities have a distinct effect on RSA depending on the nutrient types and the amount supplied or locally concentrated in the soil environment [2]. The macronutrient sulfur (S), in the form of sulfate, is a mobile resource found in deeper soil profiles [1]. It is proposed that a combination of a thick and deep primary root (PR) with few and long lateral roots (LRs) can improve the uptake of S [3]. To achieve the adjustment of RSA, individual root traits can be regulated independently in response to changes in nutrient availabilities and patterns of nutrient distributions in the soil environment [4]. Among all root traits, LRs are phenotypically evaluated by length, total numbers, and density, which is considered a major determinant of RSA [5]. Changes in sulfate availability have a variable effect on LR development. For example, several studies have demonstrated that S deficiency leads to reduction in LR length [2,6,7,8,9] and LR number or density [2,7,9]. However, active growth of LR appears to be another response to sulfate starvation as it is described with longer LR length [10,11] and higher LR number or density [11,12,13]. 

Studies on functional characterization of small signaling peptides (SSPs) reveal that several distinct groups of SSPs play important roles in plant root development [14,15,16,17,18,19,20,21,22,23,24]. Nitrogen (N)-responsive C-TERMINALLY ENCODED PEPTIDEs (CEPs) are a group of SSPs known to be functional as negative regulators of LR development under N-limited conditions [14,15,16,17], while they have also been shown to be involved in long-distance regulation of N uptake [18]. A few distinct members of the CLAVATA3 (CLV3)/EMBRYO SURROUNDING REGION (CLE) family are also well characterized as SSPs regulating LR development in *Arabidopsis thaliana* (*A. thaliana*) [19,24]. *CLE1*, *-3,* -*4,* and *-7* are characterized particularly in relation to N nutritional responses as they are expressed in roots under N deficient conditions [19]. The LR phenotypes depicted in our previous study therefore highlight the *CLE3* gene expression enhanced in roots as a potential mechanism suppressing LR development under low N supply or in response to systemic N demand signals [19]. *CLE2* and *CLE3* also demonstrate positive responses of gene expression after N resupply to N-starved seedlings, as their transcript levels are shown to dramatically increase in response to nitrate and ammonium, respectively [19,25]. *CLE1, -2, -3, -4,* and *-7* are predominantly expressed in pericycle cells in roots, while *CLE1* and *CLE5* promoters are also found to be active in epidermal cells of the primary root tip [19]. These partially overlapping expression patterns, environmental responses and feedback regulation suggest functional redundancy of these N-responsive *CLE* genes in LR development. In addition to mechanisms characterized in relation to the N status, *CLE* genes are also known to be transcriptionally modulated by changes in availability of other macronutrients including S, phosphorus (P) and potassium (K) [24,26], as well as perturbation of cellular status caused by phytohormones and environmental stimuli [24,27]. More specifically to responses to S in roots, *CLE12* and *CLE2* are known to be up- and down-regulated, respectively, by S deprivation [24]. Thus, *CLE2* seems to be controlled by the S-responsive pathways in addition to being up-regulated by resupply of N [19]. In contrast, the S-responsive regulation of *CLE3* gene expression has not been studied despite its roles in LR development documented in relation to responses to N nutrition. CLE2 peptide has been shown to physically bind to the CLE receptor CLAVATA1 (CLV1) [28], and CLE3 requires CLV1 to transmit signals to modulate LR development [19]. Based on these aspects of nutrient-responsive regulation of *CLE2* and *CLE3* gene expression and their specific relationship with CLV1, we focused on investigating the effect of S on the CLE-CLV1 signaling pathway and LR development.

Here, we report the CLE-CLV1 signaling pathway is associated with S-responsive mechanisms modulating LR development in *A. thaliana*. The results shown in this study suggest a link between the morphological responses of LRs and the CLE-CLV1 signaling pathway, as well as S-responsive regulations of *CLE2* and *CLE3* genes in *A. thaliana* seedlings exposed to prolonged S deficiency.

## 2. Results

### 2.1. CLAVATA1 Controls Lateral Root Development under S Deficiency

**Figure 1:**
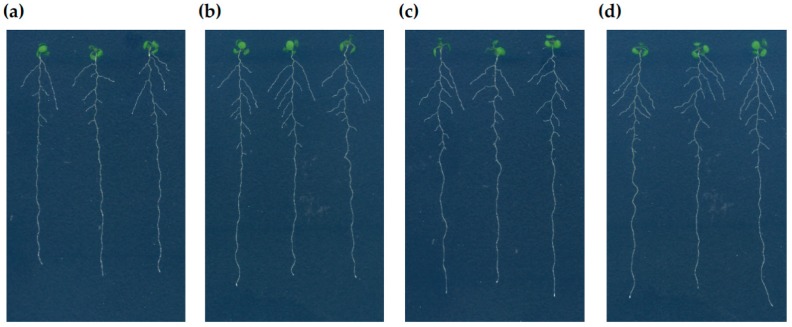
Effect of sulfur (S) supply on root phenotypes of *Arabidopsis thaliana* (*A. thaliana*) seedlings. The wild-type Columbia-0 (Col-0) seedlings were grown vertically on –S (15 μM sulfate) or +S (1500 μM sulfate) medium for 7 days and transferred to –S or +S medium in a reciprocal manner to be grown subsequently for 3 days. The scanned images of seedlings on Day 10 are shown. The images show the phenotypes of representative seedlings transferred from (**a**) –S to –S, (**b**) –S to +S, (**c**) +S to +S, and (**d**) +S to –S, respectively.

To investigate the effect of S supply on root development, the wild-type *A. thaliana* (accession Columbia-0 (Col-0)) were germinated and precultured on a –S (15 μM sulfate) or +S (1500 μM sulfate) medium for 7 days. The seedlings were then transferred to the medium with the same concentration of sulfate, or from the –S preculture to the +S medium, or from the +S preculture to the –S medium, and grown for 3 days to validate the effect of S starvation and S replenishment (Figure 1). The most significant changes in root morphology at Day 10 were the decrease in length and number of LRs after long-term limitation of S (Figure 1a; plants transferred from –S to –S) compared to the recovery of the roots observed in response to supply of sulfate (Figure 1b; plants transferred from –S to +S). The PR growth was slightly enhanced when the seedlings were transferred from the –S preculture to the +S medium (Figure 1a,b). In contrast, the seedlings from the +S preculture medium transferred either to the +S or –S medium showed no significant changes in the root morphological phenotypes (Figure 1c,d). 

**Figure 2:**
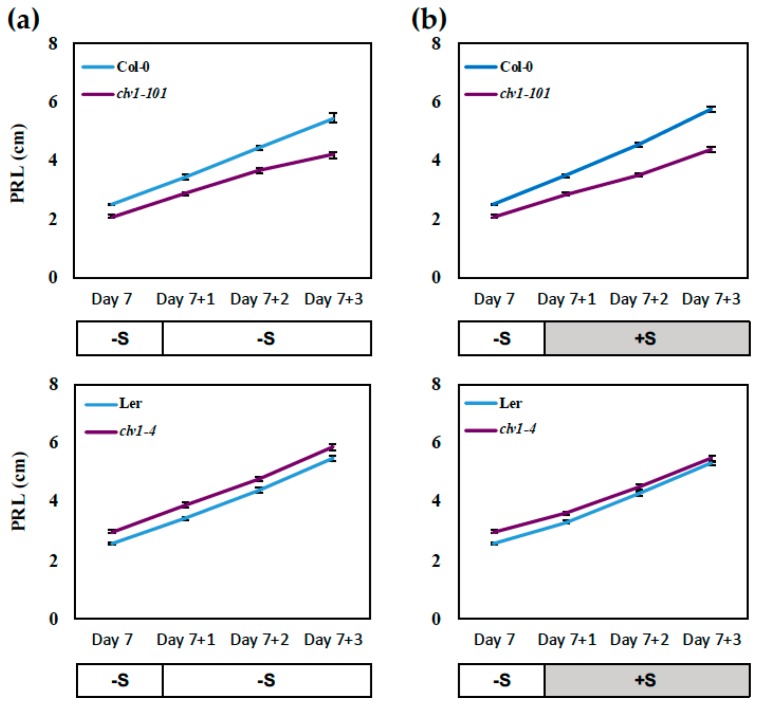
Effect of S supply on primary root growth. Wild-type (Col-0 and L*er*) and *clv1* mutant lines (*clv1-101* and *clv1-4*) were grown vertically on –S (15 μM sulfate) medium for 7 days and then transferred to (**a**) –S medium or (**b**) +S medium to be grown subsequently for 3 days. Primary root length (PRL) was measured before (Day 7) and after the transfer (Day 7+1, 7+2, and 7+3) as indicated on each graph. Values show means (± SE) of 24 individual plants per treatment. White and dark grey bars labeled –S (15 μM sulfate) and +S (1500 μM sulfate) below the graph represent the S conditions before the transfer and during the treatment.

**Figure 3:**
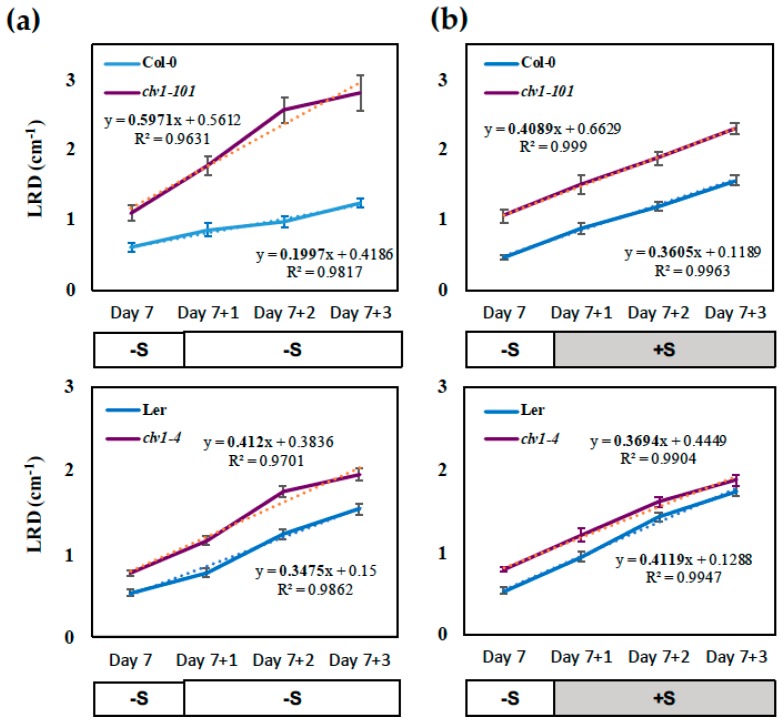
Effect of S supply on lateral root density (LRD). Wild-type (Col-0 and L*er*) and *clv1* mutant lines (*clv1-101* and *clv1-4*) were grown vertically on –S (15 μM sulfate) medium for 7 days and then transferred to (**a**) –S medium or (**b**) +S medium to be grown subsequently for 3 days. LRD was calculated at each time point based on the number of emerged lateral roots (LRs) and the length of the primary root (PR) of one seedling. Values represent means (± SE) of 24 individual plants per treatment. The equations for the linear repression and the R-squared values are indicated on each graph.

**Figure 4:**
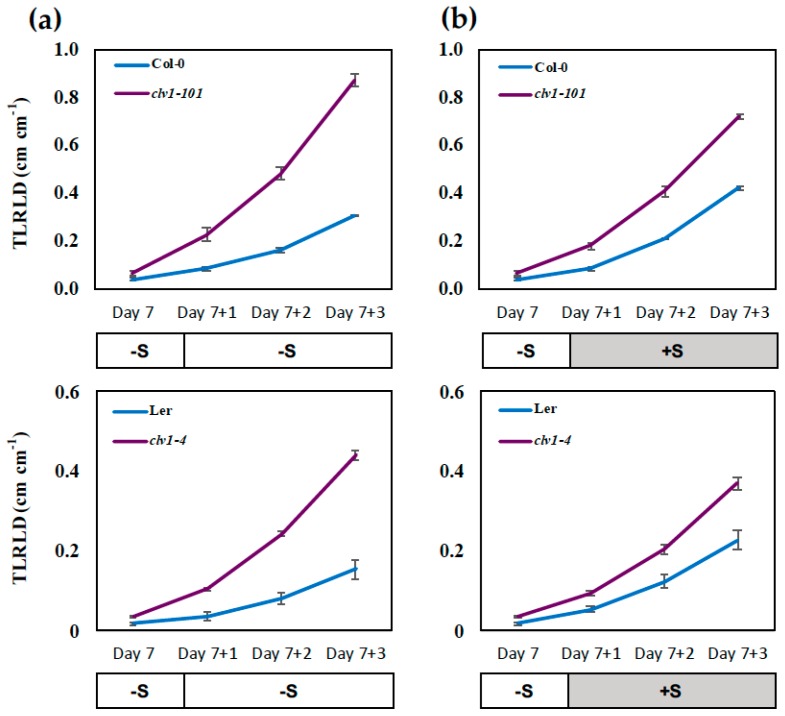
Effect of S supply on total lateral root length density (TLRLD). Wild-type (Col-0 and L*er*) and *clv1* mutant lines (*clv1-101* and *clv1-4*) were grown vertically on –S (15 μM sulfate) medium for 7 days and then transferred to (**a**) –S medium or (**b**) +S medium to be grown subsequently for 3 days. TLRLD was calculated at each time point based on the lengths of the entire LR in one seedling and the length of the PR. Values show means (± SE) of 24 individual plants per treatment.

Based on these observations, we hypothesized the CLE-CLV1 signaling pathway, which has been shown to regulate LR development under N-starved conditions [19], would be involved in pathways modulating root growth under S-limited conditions. To test this hypothesis, we focused on studying the effect of *clv1* mutations on root growth under conditions where the differences in root morphology were most significant. As mentioned, the most significant morphological changes were observed for the –S-precultured seedlings transferred to either the –S or +S medium (Figure 1a,b). The effect of S supply on changes in root morphology was recorded at Day 7 before the transfer and during the 3 consecutive days in the post-transfer growth period until Day 10 (Figure 2, Figure 3 and Figure 4). The results indicated that changes in S conditions had little influence on primary root growth (Figure 2). The *clv1-101* mutant had slightly shorter PR values compared to its background wild-type accession (Col-0), regardless of changes in S conditions; however, the same phenotype was not observed in the *clv1-4* mutant in comparison with its background wild-type accession Landsberg *erecta* (L*er*) (Figure 2). 

As for the phenotypes associated with LR development, the LR density (LRD; total lateral root number divided by primary root length (cm^−1^)) was maintained at low levels in the wild-type Col-0 seedlings during the period of S limitation which was extended for 3 days after the transfer, while it was restored upon sulfate supply and became higher during the time course (Figure 3). The LRD showed a trend of linear increase over the time course, for which the slope value can be calculated based on linear regression. The slope value of the linear regression line is expressed as the number of LR developed in one cm unit length of PR per day, indicating the daily increase rate of LRD. Thus, it provides a quantitative measure for the assessment of incremental changes in LR development demonstrated over time as a part of the RSA phenotype (Figure 1). The slope values were 0.36 on +S and 0.20 on –S medium, respectively (Figure 3a,b), suggesting that LR emergence which gave rise to visible LR happened 1.8-fold more frequently in Col-0 roots transferred to sulfate-supplied conditions compared to those exposed to prolonged S deficiency. These estimations were generally consistent with the visible phenotypes (Figure 1). Similar changes were observed in the wild-type L*er* seedling; however, the differences between the slope values (0.41 on +S and 0.35 on –S medium, respectively) were not as significant as those estimated for Col-0 (Figure 3a,b). 

Consistent with our previous findings [19], LRD was constantly higher in the *clv1* mutants than in the wild-type (Figure 3). LRD increased significantly in the *clv1* mutants during the period of prolonged S deficiency (Figure 3a; transfer from –S to –S) compared to those transferred to the sulfate-supplied medium (Figure 3b; transfer from –S to +S). In contrast, the wild-type seedlings showed a lower increase rate of LRD on the –S medium compared to those transferred to the +S medium. Based on the slope values of the linear regression, the daily increase rate of LRD was estimated to be 3-fold greater in the *clv1-101* mutant than in Col-0 under S-deficient conditions (Figure 3a). In contrast, upon sulfate supply, the slope value representing the increase rate of LRD was diminished in *clv1-101* though increased in Col-0, apparently converging to the same level (Figure 3b). Similar trends were found when the *clv1-4* mutant was compared with L*er*, while the differences were not so obvious as those shown with the *clv1-101* mutant and Col-0. These results indicated that CLV1 is a signaling component which is necessary for regulation of LR development under S-deficient conditions. 

Lateral root branching density (BD) is an alternative measure of LR density, which is calculated by the number of emerged LRs divided by the length of the branching zone (the distance from shoot base to the youngest emerged LR) [29]. BD was calculated 2 and 3 days after the transfer of seedlings to –S or +S medium from 7 days of preculture on the –S medium (Supplemental Figure S1). Consistent with the results shown for LRD (Figure 3), the wild-type plants exposed to prolonged S deficiency showed low BD, while this branching phenotype was recovered upon sulfate supply (Supplemental Figure S1). BD was significantly higher in the *clv1* mutants compared to the wild-type under prolonged S deficiency, while it was lowered after S supplementation. These changes in BD implicate that LR primordia located between the emerged LRs are partially arrested or delayed in progression in the wild-type under the prolonged S deficiency, while their growth can be recovered by the S supplementation. These results also indicate that CLV1 is involved in the regulatory pathway that inhibits the growth of LR primordia, as shown by an increase in BD in the *clv1* mutants relative to the wild-type (Supplemental Figure S1), which confirms our previous findings providing evidence for its essential role in regulating developmental stage progression of LR primordia [19]. Our present findings suggest that long-term S deficiency signals to the CLV1-directed pathway to modulate LR development as demonstrated by changes in LRD and BD. 

The total LR length density (TLRLD; total lateral root length divided by primary root length (cm cm^−1^)) was also calculated based on the measurement of the length of the entire LR developed in the root system (Figure 4). The *clv1* mutants showed a significant increase in TLRLD during the period of prolonged S limitation, where an enhanced response to –S was identified in comparison with +S. In contrast, the wild-type showed a more significant increase in TLRLD after sulfate supplementation. These opposing effects of S limitation and sulfate supply on TLRLD in the *clv1* mutants and the wild-type were consistent with those identified for LRD and BD (Figure 3 and Supplemental Figure S1). These results further suggested the essential role of CLV1 in regulation of LR development under S deficiency. As shown in Figure 1, the effect of +S to +S and +S to –S transfer on root morphology was examined simultaneously in this study. In contrast to the results obtained from the –S to –S and the –S to +S transfer experiments (Figure 3 and Figure 4), the LRD and TLRLD of the +S-precultured seedlings changed only slightly in response to S supplementation and S removal (Supplemental Figures S2 and S3). The LRD and TLRLD were higher in the *clv1* mutants than the wild-type as expected, but there was no substantial effect of S in contrast to the phenotypes identified in roots transferred from the –S preculture to –S and +S conditions (Figure 3 and Figure 4). 

### 2.2. Regulation of CLE2 and CLE3 Gene Expression under S Deficiency 

**Figure 5:**
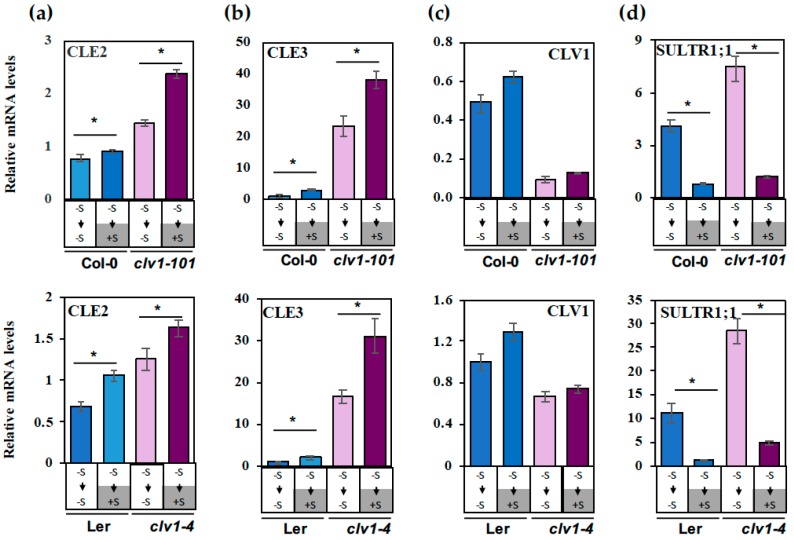
Regulation of *CLE2* and *CLE3* transcript levels by S deprivation and sulfate supply. Wild-type (Col-0 and L*er*) and *clv1* mutant lines (*clv1-101* and *clv1-4*) were grown vertically on –S (15 μM sulfate) medium for 7 days and then transferred to –S medium or +S medium to be grown subsequently for 3 days. The mRNA levels of (**a**) *CLE2,* (**b**) *CLE3,* (**c**) *CLV1* and (**d**) *SULTR1;1* in roots at Day 10 (i.e., Day 7+3) were determined by real-time PCR. Roots of wild-type plants grown on the +S medium for 10 days were used as reference samples for relative quantifications. Actin 2 and Ef1α were used as internal controls. Mean values (±SE) of 4 biological replicates with 8 plants for each replicate were calculated using two internal controls. Asterisks indicate statistically significant differences (*P* < 0.05) between gene expression on –S and +S treatment. The S conditions and the order of transfers are shown by white and dark grey bars and arrows below the graph.

To investigate the responses of *CLE* gene expression to the alteration of S nutritional status, the wild-type and *clv1* mutant lines were grown with different S supplies as indicated above for the root phenotype analysis. The root RNAs were extracted at Day 10 (i.e., Day 7+3) for the gene expression analysis. Among the *CLE* genes, *CLE2* and *CLE3* were selected for gene expression analysis, because *CLE2* was previously reported as a S-responsive *CLE* gene [24], and *CLE3* was shown to be significantly up-regulated by N deficiency and inhibits LR development through acting on the CLV1 signaling pathway [19]. *SULTR1;1* encodes a high-affinity sulfate transporter which is highly regulated in response to sulfur deficiency (–S) in the epidermis and cortex of *A. thaliana* roots [30,31]. Therefore, *SULTR1;1* was used as an indicator for tracking the changes in S status. The results indicated prolonged S deficiency (transfer from –S to –S) caused repression, although the sulfate replenishment (transfer from –S to +S) allowed induction of *CLE2* and *CLE3* gene expression in both wild-type and *clv1* mutant lines (*P* < 0.05) (Figure 5a,b). *CLE3* showed greater responses (2–3 fold) to S than *CLE2* (1.2–1.5 fold). 

To investigate the effect of S on CLV1-mediated feedback regulation, the *clv1*/wild-type ratios of the *CLE2* and *CLE3* mRNA levels were calculated and compared between the prolonged –S (transfer from –S to –S) and the sulfate supplied (transfer from –S to +S) conditions. The results indicated both the *clv1-101*/Col-0 and *clv1-4*/L*er* ratios of the *CLE3* mRNA levels under the prolonged –S conditions were higher compared to those estimated for roots transferred to +S medium, suggesting that prolonged S deficiency activates a pathway downstream of CLV1 to feedback regulate *CLE3*. In contrast to *CLE2* and *CLE3*, the *CLV1* expression levels did not change significantly by perturbation of S supply (Figure 5c). *SULTR1;1* was upregulated in roots exposed to prolonged S deficiency while repressed upon sulfate replenishment, showing typical patterns of its S-responsive gene expression, which verified that the S conditions tested in this study were appropriate (Figure 5d). 

## 3. Discussion

**Figure 6:**
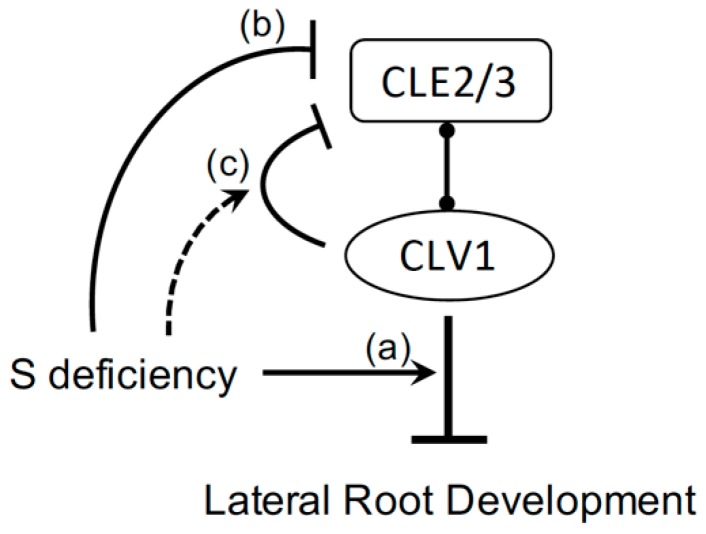
Schematic model of signaling pathway controlling lateral root development under S-deficient conditions. S availability affects the activity of the signaling module composed of CLAVATA3 (CLV3)/EMBRYO SURROUNDING REGION (CLE) peptides and CLAVATA1 (CLV1) leucine-rich repeat receptor kinase in roots. The model proposes three potential pathways involved in regulation of LR development under S-deficient conditions. Regulatory components expressed downstream of CLV1 become active under S deficiency and inhibit LR development (**a**). *CLE2* and *CLE3* can be repressed under S deficiency directly or partially through the CLV1-mediated feedback mechanism (at least for CLE3) to reduce the input of CLE signals (**b**,**c**).

The results shown in this study indicate that the CLE-CLV1 signaling pathway is involved in regulation of LR development under prolonged S deficiency. The CLE-CLV1 signaling module has direct impact on LR development and physiological responses associated with changes in RSA. The proposed model describes three steps of the S-dependent signals and their coordinated actions controlling the CLE-CLV1-dependent pathway, extending our knowledge on S nutrient signaling mechanisms involved in regulation of RSA (Figure 6). 

The inhibition of LR development occurs in the wild-type plants exposed to prolonged S deficiency, while this inhibition is abolished in the *clv1* mutants (Figure 3 and Figure 4). In contrast, transferring the seedlings to the S-sufficient medium leads to a recovery of LR development in the wild-type, but rather a diminished response in the *clv1* mutants. The increase rate of LRD calculated based on linear regression provides additional information allowing for a quantitative interpretation of the LR phenotypes, as it is shown to be altered in response to S conditions and different among the genotypes being tested in this study. As described in Figure 3, the daily increase rate of LRD was greater in the *clv1* mutant than in the wild-type during the period of prolonged S deficiency, but estimated to be similar between the *clv1* mutant and the wild-type after the sulfate supply. These findings suggest that CLV1 is a signaling component acting on pathways negatively controlling LR development. Regulatory components expressed downstream of CLV1 may be activated under S-deficient conditions (Figure 6). The number of the emerged and visible LR was counted in experiments performed in this study. Thus, we assume that an increase in LRD after the transfer of precultured seedlings to the new medium corresponds to the number of newly emerged LR, and it is associated with developmental stage transition of LR primordia—as has been shown previously [19]. The inhibition of LR development under S deficiency and its CLV1 dependency may reflect these developmental mechanisms previously shown with relevance to the N starvation responses [19]. 

*CLE2* and *CLE3* transcript levels decrease under S deficiency in both the wild-type and the *clv1* mutant lines (Figure 5). These transcript profiles suggest that *CLE2* and *CLE3* are repressed under S-deficient conditions in a CLV1-independent manner. In addition, changes in the *clv1*/wild-type ratios of the *CLE3* transcript levels indicate that the CLV1-dependent feedback control mechanism may be strengthened under S-deficient conditions (Figure 5). However, the *CLE2* and *CLE3* transcript levels being reduced in the wild-type plants under S-deficient conditions had no positive impact on promoting LR development. As shown in our model, components expressed downstream of CLV1 are suggested to be more crucial and directly involved in regulation of LR development (Figure 6). The *CLE2* and *CLE3* transcript repression occurring under S-deficient conditions and partially in conjunction with the CLV1-mediated feedback pathway may be considered mechanisms that counteractively reduce the amplitude of the input CLE signals (Figure 6). *CLE2* and *CLE3* are expressed in the pericycle, while CLV1 is expressed in the phloem companion cells [19]. *CLE2* expression is also found in the vascular tissue at the base of developing LR primordia [19,32]. It is suggested that these CLE peptides are SSPs secreted from the pericycle and diffused toward the phloem companion cells to bind to the receptor CLV1, and the CLV1-downstream components expressed in the phloem possibly carry the information to regulate development of LR primordia in long distance via trafficking through the phloem connection [19,33]. The CLE3-CLV1 ligand–receptor relationship and the potential long-distance effect of this signaling module are supported by evidence showing strong inhibition of LR development in transgenic lines with *CLE3* gene overexpression driven by its own promoter in the wild type background and an apparent insensitive response to the same transgene overexpression observed in the *clv1* mutant [19]. Our previous findings provide further implication that additional CLV1-downstream signals may be present and in turn sent back to the pericycle to feedback control *CLE* gene expression [19,33]. According to these models, both CLE peptides and CLV1-downstream components are likely transported between the two different cell types, i.e., pericycle and phloem, and through the vascular system. The actual signals involved in these putative long distance pathways yet remain to be identified.

## 4. Materials and Methods 

### 4.1. Plant Growth Conditions

Two *Arabidopsis thaliana* accessions, Columbia-0 (Col-0) and Landsberg *erecta* (L*er*), and their corresponding *clv1* mutants, *clv1-101* and *clv1-4* were used in this study. Plants were grown vertically on a nutrient medium containing 1% agar and 1% sucrose as described previously in Reference [19] in growth chambers (CU-36L4; Percival Scientific, Perry, IA, USA) conditioned at 22 °C under 16h-light/8h-dark long-day cycles with 75 µmol m^−2^ s^−1^ light intensity. Agar was washed 6 times with 1 liter of deionized water and remaining water was poured off after each agar settlement to remove sulfate. S-replete (+S) medium contained 1500 μM MgSO_4_ as the sulfur source. S-deficient (–S) medium contained 15 μM MgSO_4_, and Mg concentration was adjusted to 1500 μM by adding MgCl_2_. After 7 d preculture on +S or –S medium, the seedlings were transferred to plates with the same or different concentration of sulfate and grown for 3 d.

### 4.2. Root Analysis

Roots on agar plates were scanned at Day 7 before transfer and the following 3 days after transfer by using a scanner (Epson Perfection V700 Photo; Seiko Epson, Suwa, Japan) at 300 dpi. The images of roots were traced and measured using ImageJ. LR number, LR length, and PR length were recorded for calculation of LR density (LRD, cm^−1^) and total LR length density (TLRLD, cm cm^−1^). Total LR length (TLRL, cm) is the sum of the entire lateral root length per plant. 

### 4.3. Quantitative Real-Time PCR 

Roots were homogenized by using TissueLyser II (Qiagen, Hilden, Germany). Total RNAs were extracted by using E.Z.N.A. ^®^ Plant RNA Kit (Omega Bio-Tek, Norcross, GA, USA). Turbo DNA-free kit (Invitrogen, Thermo Fisher Scientific, Waltham, MA, USA) was used for genomic DNA removal of RNA samples. First-strand complementary DNA (cDNA) was prepared from 500 ng of root total RNA by using SuperScript III First-Strand Synthesis System (Invitrogen, Thermo Fisher Scientific). Quantitative real-time PCR was performed by using SYBR Select Master Mix (Applied Biosystems, Thermo Fisher Scientific) on a QuantStudio 7 Flex Real-Time PCR System at the Research Technology Support Facility (RTSF) of Michigan State University. The primers were previously published [19,31,34].

## Supplementary Materials

The following are available online at https://www.mdpi.com/2223-7747/8/4/103/s1, Figure S1: Effect of S supply on lateral root branching density, Figure S2: Effect of S removal on lateral root density (LRD), Figure S3: Effect of S removal on total lateral root length density (TLRLD).
