# Supplementary material for: CLE-CLAVATA1 Signaling Pathway Modulates Lateral Root Development under Sulfur Deficiency
Source: Plants (Basel). 2019 Apr 18;8(4):103. doi: 10.3390/plants8040103 (PMC6524044; doi:10.3390/plants8040103)
Supplement: Supplementary file 1 [file plants-08-00103-s001.pdf]

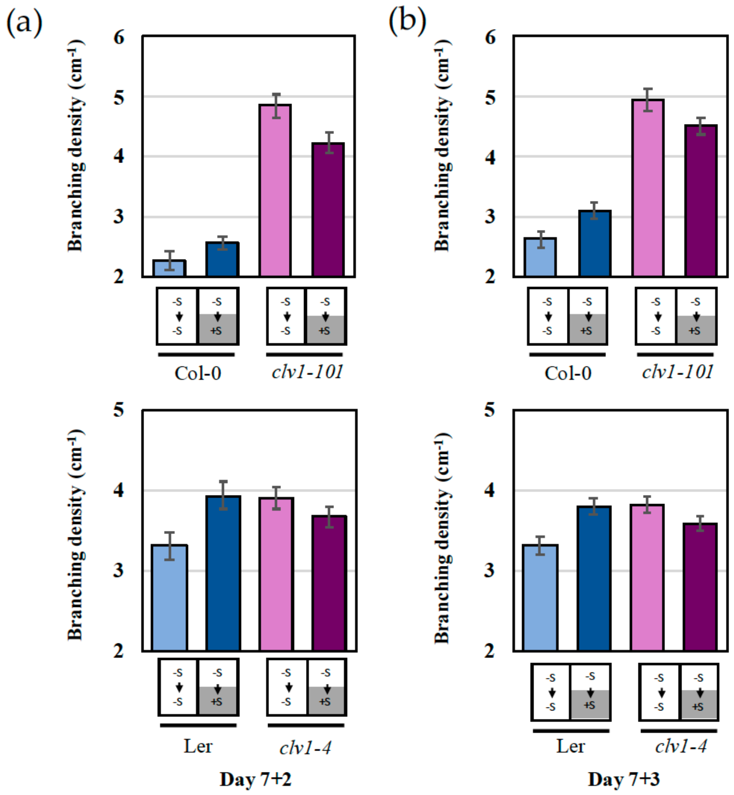


**Supplemental Figure 1.** Effect of S supply on lateral root branching density. Wild-type (Col-0 and L*er*) and *clv1* mutant lines (*clv1-101* and *clv1-4*) were grown vertically on -S (15 µM sulfate) medium for 7 days and then transferred to +S medium or -S medium to be grown subsequently for 3 days. Branching density (BD) is the number of emerged LRs divided by the length of the branching zone (cm). BD at differing S condition at Day 7+2 (**a**) and Day 7+3 (**b**), respectively, are shown. Values show means (± SE) of 24 individual plants per treatment. The S conditions and the order of transfers are shown by white and dark grey bars and arrows below the graph.

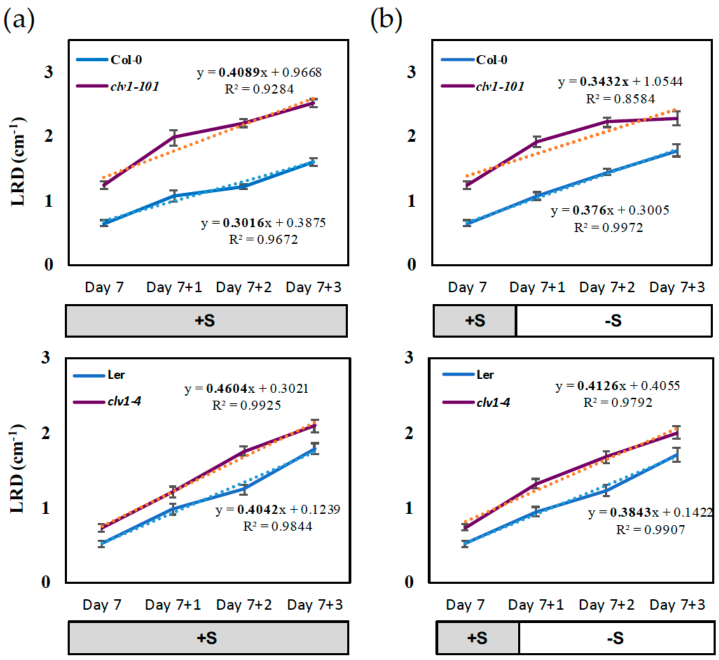


**Supplemental Figure 2.** Effect of S removal on lateral root density (LRD). Wild-type (Col-0 and L*er*) and *clv1* mutant lines (*clv1-101* and *clv1-4*) were grown vertically on +S (1500 µM sulfate) medium for 7 days and then transferred to (**a**) +S medium or (**b**) -S medium to be grown subsequently for 3 days. LRD was calculated at each time point based on the number of emerged LR and the length of PR of one seedling. Values represent means (± SE) of 24 individual plants per treatment. The equations for the linear repression and the R-square values are indicated on each graph.

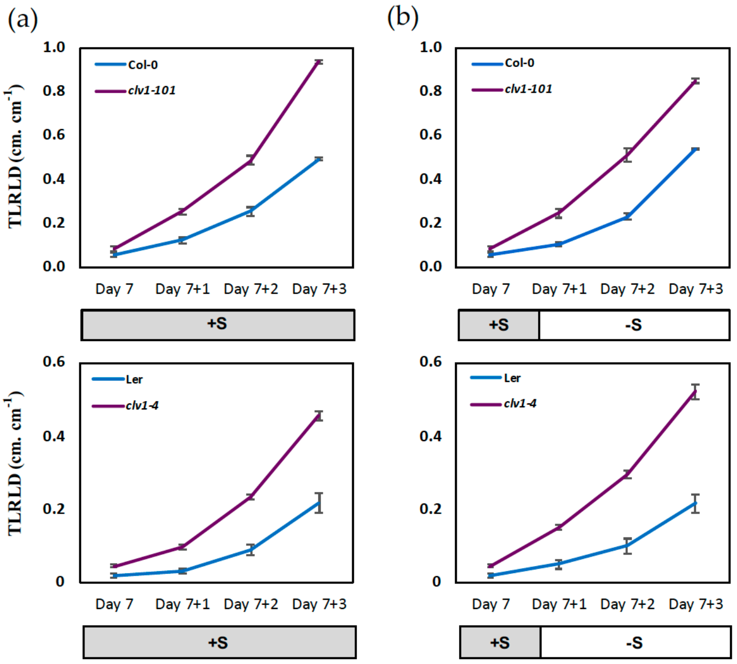


**Supplemental Figure 3.** Effect of S removal on total lateral root length density (TLRLD). Wild-type (Col-0 and L*er*) and *clv1* mutant lines (*clv1-101* and *clv1-4*) were grown vertically on +S (1500 μM sulfate) medium for 7 days and then transferred to (**a**) +S medium or (**b**) -S medium to be grown subsequently for 3 days. TLRLD was calculated at each time point based on the lengths of the entire LR in one seedling and the length of the PR. Values show means (± SE) of 24 individual plants per treatment.
